# Non-Invasive Wearable Technology to Predict Heart Failure Decompensation

**DOI:** 10.3390/jcm14207423

**Published:** 2025-10-21

**Authors:** Jack Devin, Eden Powell, Dylan McGagh, Tyler Jones, Brian Wang, Pierre Le Page, Andrew J. M. Lewis, Oliver J. Rider, Andrew R. J. Mitchell, John A. Henry

**Affiliations:** 1 Cardiology Department, Jersey General Hospital, St Helier, Jersey JE1 3QS, UK; j.devin@health.gov.je (J.D.); eden.powell@spc.ox.ac.uk (E.P.); t.jones@health.gov.je (T.J.); brianxwang@hotmail.co.uk (B.W.); p.lepage@gov.je (P.L.P.); an.mitchell@health.gov.je (A.R.J.M.); 2 Medical School, Medical Sciences Division, John Radcliffe Hospital, University of Oxford, Oxford OX3 9DU, UK; 3 Nuffield Department of Population Health, University of Oxford, Oxford OX3 7LF, UK; dylan.mcgagh@ndorms.ox.ac.uk; 4 Oxford Centre for Clinical Magnetic Resonance Research, Division of Cardiovascular Medicine, Radcliffe Department of Medicine, University of Oxford, Oxford OX3 9DU, UK; andrew.lewis@cardiov.ox.ac.uk (A.J.M.L.); oliver.rider@cardiov.ox.ac.uk (O.J.R.)

**Keywords:** wearables, heart failure, sensors, digital health, decompensation

## Abstract

Heart failure (HF) remains a leading cause of recurrent hospitalisations worldwide, largely driven by acute episodes of decompensation. Early identification of impending decompensation could enable timely intervention and potentially prevent costly admissions. Non-invasive wearable devices have emerged as promising tools for continuously monitoring physiological parameters and detecting early signs of deterioration. This review summarises recent advances in wearable technologies designed to predict HF decompensation and appraises their ability to generate clinically useful alerts. It will examine various modalities designed to monitor different aspects of cardiorespiratory physiology that have the potential to detect abnormalities preceding heart failure decompensation. Broadly, these devices either monitor physical activity capacity and cardiac function or monitor changes in pulmonary fluid congestion. We will also cover evidence exploring whether these devices can generate timely alerts for interventions to improve patient outcomes and reduce hospitalisations. However, despite advances in these technologies, challenges remain regarding their accuracy and usability for remote monitoring, as well as concerns with data storage, processing, patient adherence, and integration into existing healthcare workflows. While current limitations exist, previous results warrant further research into this area, with a focus on larger randomised trials, exploring both single- and multi-sensor systems, using artificial intelligence and cost-effectiveness analysis. Overall, non-invasive wearables represent an opportunity to create a more proactive approach to HF management, with the potential to shift the paradigm from reactive treatment to anticipatory care.

## 1. Introduction

Heart failure (HF) affects more than 64.3 million people globally [1] and is characterised by substantial morbidity, mortality and a high frequency of hospitalisations, which in turn place a considerable strain on healthcare resources [2].

Episodes of acute decompensation of HF are the main driver of admissions, however, the conventional methods for patient monitoring, which primarily relies on symptom reporting, daily weight measurements and vital measurements, have been shown to have variable benefit in forecasting and reducing episodes of decompensation, with some large trials showing no added benefit in avoiding hospitalisations [3,4]. Similarly, the presence of subjective shortness of breath and estimated oedema and jugular venous pressures do not correlate well with pulmonary capillary wedge pressure and thoracic bioimpedance as measurements of congestion [5]. On the other hand, multiple meta-analyses [6,7,8] have shown overall reduced hospitalisations with remote monitoring, and similar benefits were also seen in the TIM-HF2 trial, of which a unique element alongside telemonitoring with conventional methods was a 24/7 emergency call centre [9,10].

There has been a recent rapid expansion in the wearable-based digital health technologies (DHTs) market and consequential interest in the use of non-invasive wearable devices in HF care in the context of remote monitoring (RM). These external devices, which range from smartwatches and fitness trackers to vests and adhesive patches, offer the opportunity for continuous, passive monitoring of physiological parameters while a patient is out of the hospital. Through tracking of parameters such as physical activity, as well as physical measurements such as heart rate and respiratory rate, these technologies offer the theoretical ability to detect subtle and gradual deterioration, which may precede acute decompensation (Table 1).

In this narrative review, we examine the physiological rationale and current landscape of wearable-based DHTs in predicting HF decompensation. We summarise the key studies to date by modality, appraise their findings, and discuss the practical limitations alongside the future directions for integrating wearable-based DHTs into HF management guidelines, which currently have little endorsement of the use of wearable devices [11].

## 2. Physiological Changes Ahead of Readmission, and the Basis for Wearables

Heart failure decompensation is characterised by increased congestion, resulting in peripheral and pulmonary oedema. Patients often demonstrate congestion-related symptoms on average 3–7 days prior to hospitalisation [12], with nearly 50% of patients having symptoms present for more than 15 days prior to hospitalisation [13].

Once admitted, average length of stay is 5 to 10 days [14], with an inpatient mortality rate of up to 10% [15]. The outlook of patients living with HF following discharge is also poor with nearly 25% facing readmission in the first 30 days after discharge [16] and a 1-year mortality of over one third [17].

Importantly, the physiological changes underlying congestion, such as rising resting heart rate, reduced heart rate variability, falling activity levels, and early shifts in respiratory pattern or fluid balance (Table 2), often occur days before overt symptoms. By continuously and passively monitoring these parameters, wearable-based DHTs may detect subtle deviations from an individual’s baseline, providing timely alerts that could enable earlier intervention and adjustment of therapy than is possible with conventional clinic-based assessments.

### 2.1. Accelerometry in Predicting Decompensation

Accelerometers are widely used to quantify physical activity, detecting movement by measuring changes in acceleration across one or more axes. Detection of acceleration is useful as it reflects the intensity of an exercise and can respond to gravity, giving insight into posture and gait. Commonly integrated into wearable devices such as smart watches, vests and belts, this technology is promising. Continuous recordings of physical activity can provide a clinician with a more accurate and complete view, with the theoretical promise of allowing smaller changes over time to be identified in comparison to traditional checkups. As such, accelerometers show promise in predicting HF decompensation through the detection of changes in physical activity, which reflect worsening of symptoms associated with HF decompensation. While many studies focus on accelerometry-based HF prognostication, detecting changes in exercise intolerance as a result of fluid overload and breathlessness [34] may give these studies credence in support of accelerometers’ detection of HF decompensation.

#### 2.1.1. Step Count

Step count, derived from accelerometer data, provides a simple but informative measure of daily physical activity. There are several studies noting correlations between decreased step count and HF diagnosis and prognosis. For example, a Japanese study using a uniaxial belt accelerometer to monitor step count in patients with HF showed that step counts under 4889 steps per day were an accurate indicator of cardiac-related mortality [18]. A recent pilot study found that individuals newly diagnosed with HF showed a decline in daily step counts in the 6 months prior to diagnosis, when compared to matched controls [35]. Together, these findings suggest that reductions in step count may signal deterioration in cardiac function and could help identify patients at increased risk of decompensation.

It is also important that such devices are both feasible and acceptable in heart failure populations. In a rural US cohort, a waist-worn accelerometer provided reliable measures of habitual activity, with average wear times exceeding 15 h per day [19]. Nonetheless, only about half of participants achieved full protocol compliance, and adherence declined over time. Interviews highlighted challenges including discomfort, skin irritation, disruption of daily routines, and difficulty during illness or hospitalisation. Lower compliance was associated with older age, higher BNP, and rehospitalisation. These findings suggest that, while accelerometer monitoring is feasible and produces reliable data, acceptability and adherence are important considerations for the wider adoption of wearable monitoring in HF.

#### 2.1.2. Beyond Step Count

Accelerometer-derived metrics extend beyond simple step counts and can capture a broader profile of physical activity. For instance, distinguishing sedentary from non-sedentary time and estimating exercise intensity. Analysis of data from over 89,000 participants in the UK Biobank demonstrated that, even among individuals meeting standard physical activity guidelines, those spending more than ~10.6 h per day sedentary had a significantly higher risk of developing heart failure [20]. Similarly, another study showed that individuals at the time of HF diagnosis had a modest reduction in the daily minutes they spent very active or fairly active (62 vs. 53 min) [35]. These findings highlight the prognostic potential of accelerometer-derived measures beyond step count, particularly sedentary time. Whether such metrics can also predict decompensation among those already living with heart failure, however, remains uncertain.

Accelerometers have also been used in patients with HF post discharge from hospital. In a cohort assessed 10–14 days after admission for acute decompensated HF, patients spent only 9% of their awake time in non-sedentary behaviour, compared with 27% in stable chronic HF and 34% in healthy controls [21]. This marked reduction underscores how acute decompensation is characterised by profound inactivity, suggesting that tracking changes in sedentary and active time may provide an additional avenue for early identification of patients at risk of recurrent events.

Extending beyond descriptive associations with sedentary time and activity levels, accelerometer-derived metrics have also been directly linked to underlying cardiac pathophysiology. In a physiological study of patients with stable HFrEF, daily activity quantified by accelerometry was associated with invasive haemodynamic responses during exercise. Patients with lower habitual activity demonstrated markedly reduced stroke volume reserve and a blunted increase in cardiac index, despite similar pulmonary capillary wedge pressures [36]. These findings suggest that accelerometer outputs reflect not only behaviour but also central haemodynamic capacity, providing mechanistic support for their use in predicting physiological changes prior to a decompensation event.

Actigraphy can also be used to derive rest–activity rhythms, providing information on the strength, stability, and fragmentation of circadian patterns. In a prospective study of patients living with HF and comorbid insomnia, weaker circadian rhythm strength and lower day-to-day stability were independently associated with earlier hospitalisations and emergency department visits, even after adjusting for conventional clinical predictors [37]. These findings highlight that disruption of rest–activity rhythms may represent another accelerometer-derived marker of vulnerability, extending beyond measures of activity volume or intensity, and could offer additional prognostic value in identifying patients at risk of decompensation.

More exploratory accelerometer-derived metrics have also been investigated, such as skewness and kurtosis, which describe the distribution of activity intensity over time. Skewness reflects the asymmetry of activity patterns, for example, whether activity is concentrated in short bursts or spread evenly, while kurtosis reflects the peakedness of the distribution, with higher values indicating activity dominated by a few extreme episodes. In a study of patients with advanced chronic HF, skewness of the most active three-hour period was independently associated with all-cause mortality, improving discrimination beyond the Heart Failure Survival Score [38]. These findings highlight that variability in activity patterns, rather than total activity alone, may carry prognostic value and could represent another dimension for predicting vulnerability to decompensation.

While not yet explored in HF, emerging methodologies may improve the prognostic ability of wearable devices. One such area, compositional data analysis (CoDA), takes a summary total for the different activity behaviours (sedentary periods, sleep–wake time, light and moderate-to-vigorous physical activity) and models the change in these behaviours associated with an outcome of interest [39]. In the case of HF exacerbation, time spent in light or moderate-to-vigorous physical activity may be substituted for more sedentary time or fragmented sleep secondary to dyspnoea, altering the ratios of these data vectors within the model. This can be applied longitudinally using multi-level CoDA, where, for example, changes in symptom scores could be explored against the activity composition for that day. Another emerging area is functional data analysis, which treats individual samples as curves over a continuum rather than discrete points [40]. This may enable prediction of an event (i.e., HF exacerbation) from a long time-series of potential ‘normal’ data. However, infinite dimensionality and misalignment of parameters and overfitting of models remain major challenges.

Accelerometers can also be combined with gyroscopes and magnetometers to create inertial measurement units (IMUs), which capture postural stability and gait dynamics in addition to activity volume. In a study of older inpatients with HF, IMU-derived mobility parameters were examined during balance and gait tasks. Of the digital mobility outcomes assessed, sway path during semi-tandem stance was most strongly associated with NYHA class, with more severe HF linked to a reduced sway path [41]. This suggests that wearable-based DHTs can detect subtle alterations in posture and gait linked to disease severity, extending beyond activity counts and offering another potential avenue for identifying patients at higher risk of functional decline or decompensation.

#### 2.1.3. Accelerometer-Derived Metrics in Clinical Trials

Accelerometers are increasingly being incorporated into HF clinical trials, where they provide objective, continuous measures of physical activity alongside traditional endpoints. An analysis of the DETERMINE trial using a waist-worn triaxial accelerometer revealed increases not only in step count but also in minutes of moderate-to-vigorous activity and vector magnitude units (VMUs) following treatment with dapagliflozin [42]. Importantly, such improvements were not identified by the six-minute walk test (6MWT), the traditional gold-standard measure of exercise capacity in heart failure.

Other clinical trials have also utilised accelerometer-derived measures of physical activity to assess intervention effects in HF. In the NEAT-HFpEF trial, a belt-worn triaxial accelerometer was used to quantify daily activity (average daily accelerometer units and hours active per day). Activity levels correlated with conventional severity indices at baseline but were dose-dependently reduced by isosorbide mononitrate, even though standard trial endpoints such as NYHA class, 6MWT, and NT-proBNP were unchanged [43]. In the VITALITY-HFpEF substudy, a wrist-worn accelerometer (ActiGraph GT9X Link) captured multiple dimensions of physical activity, including step count, energy expenditure, METs, sedentary time, and total activity counts across the most active periods of the day. However, changes in these measures did not correlate with improvements in KCCQ-Physical Limitation Score or 6MWT over 24 weeks [44]. By contrast, the WATCHFUL trial employed accelerometers as part of a structured walking intervention in HFrEF, with step-count feedback and motivational messaging. This approach increased daily step counts but did not translate into improvements in NT-proBNP or 6MWT [45]. Collectively, these studies demonstrate not only the feasibility of accelerometer deployment in HF trials but also their potential to generate novel endpoints, raising the possibility that such measures could one day inform risk stratification and prediction of decompensation.

#### 2.1.4. Challenges and Limitations of Accelerometry

Although monitoring physical activity with accelerometers has shown promise in identifying patients at risk of poorer outcomes and may hold potential for predicting heart failure decompensation, several practical challenges and limitations need to be considered. A major drawback is the lack of standardisation between devices in terms of algorithms and estimates of physical activity. Fokkema et al. [46] tested various wearable devices capable of measuring step count at different walking speeds and found there to be significant variability between devices and speeds when compared against manual hand counting (the gold standard for measuring step count). This is likely related to inherent device-based errors, with validation studies reporting intraclass correlation coefficients of around 0.80 [47,48]. This is likely to be important when trying to detect a signal of deterioration in a clinical context. Moreover, raw accelerometer outputs require complex processing, and there is no consensus on optimal thresholds or which derived metrics—such as sedentary time, minutes of moderate-to-vigorous activity, or vector magnitude units—are most informative for clinical use.

In addition to device type, the location of measurement is important. Wrist-worn or uniaxial accelerometers may be less sensitive when capturing true physical activity than omnidirectional/triaxial accelerometers worn at the waist. One study showed that ankle-worn accelerometers were accurate in estimating step counts but, importantly, had a lower mean absolute percentage error (MAPE) than a waist-worn device (<10%) [49]. Some uniaxial devices struggle to detect dynamic or non-walking movements, and wrist-worn devices may misclassify upper-limb activity as steps, leading to overestimates of physical activity and energy expenditure [50]. As such, waist-worn accelerometers may have a greater specificity and sensitivity for sedentary behaviour compared to wrist-worn devices [51]. However, adherence with waist-worn devices is often lower, as they are removed for showering or sleeping and may be forgotten. In older patients with HF, the improved compliance and acceptability of wrist-worn devices may outweigh their relative loss of accuracy.

Even when devices are worn correctly, physical activity is influenced by many factors other than HF status, including musculoskeletal comorbidities, chronic lung disease, depression, or environmental factors such as weather. This lack of specificity complicates the interpretation of reduced activity as an early sign of decompensation.

Addressing these challenges including technical variability, patient compliance, non-cardiac confounders, and clinical integration will be essential before accelerometers can be translated from research tools into reliable instruments for predicting heart failure decompensation.

## 3. Electromechanical Changes in Predicting Decompensation

In HF, the mechanical dysfunction and changes in electrical conductivity of the heart differ in the decompensated state, providing an opportunity to monitor for differences between patients with stable chronic HF and decompensation.

As the cardiovascular system becomes unable to compensate, the variability in the R-R interval changes, decreasing heart rate variability (HRV) as sympathetic tone increases in response to reduced cardiac output. Low HRV has been shown to predict the development of incident HFpEF in the Women’s Health Initiative cohort [52], to predict 1-year mortality in patients with established HFpEF [53], and to be modifiable with exercise training in HFpEF, where improvements in HRV paralleled gains in functional capacity [54]. Whether HRV data gained from wearable-based DHTs could help predict HF decompensation is unknown.

### 3.1. Photoplethysmography (PPG)

Most wrist-worn wearables use photoplethysomography (PPG) to assess HRV, whereby an infrared light source and detector can monitor changes in blood volume. The amount of light reflected is proportional to blood volume, and alterations in the frequencies reflected represent differences in absorbance of light between oxy- and deoxyhaemoglobin [55]. This facilitates PPG to estimate HR, HRV, and oxygen saturation. There is also research into extracting further valuable information from PPG, including respiratory rate and vascular ageing, which may lead to novel measuring techniques which are cheap and non-invasive [56,57].

While relatively affordable and accessible [58], PPG has a major limitation: it is vulnerable to motion artefacts, compromising its accuracy during exercise. Incorporating accelerometers, AI, and predictive model algorithms to predict movement can help reduce noise, improving the accuracy of PPG [59]. Despite advances in PPG technology, its ability to monitor HRV reliably in a continual remote monitoring setting has yet to be proven. In a recent comparison to ECG, Zia et al. [60] demonstrated limitations in PPG data coverage during active daytime hours, with variations according to posture and activity type. Despite this, PPG was used in the Apple Heart Study to monitor pulse intervals to identify variations indicative of atrial fibrillation. This large-scale study supports the use of PPG for automatic public monitoring of heart health. In a pilot study Shah et al. [22] PPG was used to differentiate between HF patients and non-HF patients (HFrEF vs. non-HF AUC = 0.92; and HFpEF vs. non-HF AUC = 0.85), but there was no significant difference between acute decompensated HF and chronic stable HF. While PPG failed to detect differences between these patients, there may be a significant change in HRV as a patient transitions from stable to decompensated HF, which could be detected by PPG. However, further research needs to be conducted to verify whether PPG can identify HRV changes associated with this clinical decline.

#### Future Directions of PPG

Despite limited research in the application of PPG for detecting HF decompensation, future applications of PPG may allow novel features to be extracted, potentially increasing the prognostic ability of this technology. These include calculation of blood pressure, using the second derivative of PPG to estimate vascular ageing and arterial stiffness, and tracking of sleep parameters indicative of decompensation events.

Since the PPG wave is responsive to changes in intravascular volume, it is also correlated with arterial blood pressure, with several features capable of estimating systolic and diastolic blood pressure [61]. Beyond this, the first derivative of the PPG (dPPG) captures the rate of change of the signal, reflecting the velocity of blood flow during systolic upstroke and diastolic downstroke, while the second derivative (sdPPG) describes the acceleration of the waveform and highlights inflection points that are sensitive to arterial stiffness and vascular compliance. In heart failure, where decompensation is characterised by shifts in intravascular volume, rising filling pressures, and impaired vascular compliance, these derivative features may provide early markers of haemodynamic deterioration and could support the prediction of decompensation when combined with other wearable-derived signals.

PPG can also be used to derive circadian rhythm metrics from continuous heart rate monitoring. In a recent study of patients following discharge for acute decompensated HF, cosinor analysis of wrist-worn PPG data demonstrated changes in mesor (rhythm-adjusted mean), amplitude (extent of oscillation), and acrophase (timing of peak) in the weeks preceding clinical decompensation [62]. In particular, a machine learning algorithm using these circadian metrics performed moderately well in predicting HF decompensation (AUC ~ 0.74). These findings suggest that circadian features of heart rate derived from PPG may provide early warning of deterioration, complementing conventional measures of HR and HRV.

### 3.2. Electrocardiogram (ECG)

The traditional method of measuring the electrical activity of the heart, namely the ECG, has also shown promise in predicting HF decompensation. Studies interrogating the ECG in HF show several observations: patients with HFpEF have a higher incidence of atrial fibrillation, while conversely patients with HFrEF had prolonged PR intervals, abnormal Q waves, and prolonged QRS complexes [63]. Moreover, pulmonary congestion in decompensated HF can alter the electrical resistivity of the chest, altering the ECG trace [64]. Similarly, patients with acute HF rarely have a normal ECG [65], with some characteristics such as a decreased QRS amplitude predicting worsening HF [64]. One study utilised 5 min ECG recordings via a mobile phone, calculating the interval between T-wave peak and end of T-wave (Te), observing significant correlations with NT-proBNP levels and accuracy in predicting worsening HF and 30-day mortality in decompensated patients with HF [23]. Furthermore, T-wave alternans, a marker of ventricular repolarisation instability, has shown promise in predicting arrhythmic death in HF [66]. However, its ability to detect decompensation is unknown and requires further evaluation. Therefore, with a wearable ECG, it may be possible to predict acute decompensation through monitoring changes to the wave profile if these changes can also be validated in a single lead ECG as seen in most wearable-based DHTs.

Wearable cardioverter-defibrillators have also been used to assess electrical changes in the heart. The ZOLL LifeVest has been used to monitor changes in nocturnal resting heart rate (RHR), observing that a >5 beats per minute rise in RHR more than doubled cardiovascular hospitalisation and mortality risk. The authors suggest that wearable continuous monitors recording nocturnal resting heart rate could predict these cardiac events, providing the opportunity to intervene and take preventative action [67]. Moreover, analysing trends in resting heart rate my offer personalised alerts preceding decompensation, as resting heart rate rises.

Another wearable-based DHT, an armband ECG, performed comparatively with a Holter device in HRV analysis [68]. This device also utilises an accelerometer and electromyogram to record and subsequently remove motion artefacts and electrical noise from limb muscular contraction. While superior in comfort and patient compliance, ECG traces from these devices should be analysed with caution as traces from different brands of wearable-based DHTs may vary in accuracy, as compared to conventional 12-lead ECG or Holter ECG [69].

Recently, Hasumi et al. [24] used convolutional neural network algorithms to detect HF severity from single-lead ECG recordings obtained from devices such as the Apple Watch, achieving 91.6% accuracy in distinguishing between NYHA I–II and NYHA III–IV. In addition, the authors developed the HF-index, a continuous score derived from ECG features, which showed a strong positive correlation with BNP levels and reflected changes in clinical status over time. In longitudinal monitoring, the HF-index mirrored BNP and clinical course in exemplar cases during periods that included hospitalisation.

While previous ECG studies have not explicitly focused on predicting HF decompensation, Pan et al. [70] demonstrated the feasibility of this approach using a single-channel ECG recorded from a wearable vest. Their deep learning algorithm achieved a high accuracy for identifying acute decompensated HF. Although trained on a relatively small cohort, these findings provide proof of concept that single-channel ECG signals can be leveraged to detect decompensation, supporting their potential application in at-home monitoring.

### 3.3. Mechanocardiography (MCG)

Detecting the mechanical events of the cardiac cycle provides an alternative method of monitoring heart function. This can either be performed by mechanocardiography (MCG), which includes the detection of linear acceleration (seismocardiography, SCG) and angular acceleration (gyrocardiography, GCG), or phonocardiography, which utilises microphones to detect the audible mechanical events of the heart (valve closing).

MCG utilises micro three-axis accelerometers and gyroscopes to detect microvibrations induced by cardiac movement and haemodynamics in blood vessels. The majority of research into MCG and HF uses a single accelerometer on the sternum; however, the addition of multiple accelerometers across the chest in conjunction with ECG traces improves resolution and detection of numerous cardiac time intervals [71]. Correlations between electrical and mechanical activity of the heart provide insight into cardiac function and hence many MCG devices will also utilise ECG.

Koivisto et al. [25] analysed mechanical cardiography (MCG) recordings at admission and discharge for acute HF, identifying three key features that tracked disease progression: increased root-mean-square (RMS) signal strength from SCG/GCG, reflecting greater mechanical effort of the heart; increased intensity of the third heart sound (S3); and reduced stability of the first heart sound (S1). These findings are consistent with current understanding of HF decompensation, where declining efficiency and compliance lead to stronger vibrations and altered valve function. While this study did not evaluate MCG as a continuous wearable tool, the authors proposed its potential use for patient self-screening through serial measurements. Further studies are needed to determine whether serial or continuous monitoring provides the greater value for detecting early HF deterioration.

It has been demonstrated that MCG with ECG can differentiate between decompensated and compensated patients with HF [72]. By plotting MCG graphs of cardiac movement at rest and during recovery following a 6 min walk test, they observed that the graph similarity score (GSS) is significantly higher in decompensated versus compensated patients. This relates to decompensated patients having less cardiovascular reserve and hence are not able to modulate their cardiac contractility as effectively as compensated patients during exercise, so their pre- and post-exercise MCGs are more similar. This study [72] also demonstrated that, between admission and discharge following treatment, decompensated patients had a decrease in their GSS, whereas performance in other HF metrics, such as 6MWT and heart rate response, did not show significant improvement. Monitoring of GSS may therefore provide a way for MCG to remotely predict changes in cardiac reserve, indicating the onset of HF decompensation.

MCG data has also been validated against invasive right heart catheterisation [73], observing good accuracy with MCG for estimating pulmonary artery mean pressure and pulmonary capillary wedge pressure in the validation set. The authors demonstrated the use of MCG in estimating changes in intracardiac haemodynamics, validating the accuracy of this technology.

Although MCG has not yet been applied routinely for the prediction of HF decompensation, existing studies demonstrate measurable differences between compensated and decompensated states and show correlations with invasive haemodynamics. These findings suggest that MCG, particularly when combined with ECG, could provide a non-invasive means of tracking cardiac reserve and haemodynamic stress. Further validation in longitudinal studies is needed to determine whether MCG-derived metrics can reliably detect early decompensation and be integrated into remote monitoring pathways.

### 3.4. Phonocardiogram (PCG)

A phonocardiogram describes the use of microphones or an electrical stethoscope capable of transducing the mechanical vibrations caused by heart movements. This provides the clinician with a graphical representation through which normal heart sounds can be visualised and murmurs or other additional heart sounds identified [74]. When used in combination with an ECG, it provides a representation of the mechanoelectrical function of the heart.

For example, PCG can detect the abnormal S3 heart sound, indicative of rapid ventricular filling in a dilated, volume-overloaded left ventricle [75]. Collins et al. [76] explored the use of PCG in emergency department patients with acute dyspnoea, showing improved clinical confidence but no improvement in the accuracy of diagnosis of acute decompensation. Such findings are expected as the absence of S3 does not rule out decompensation (low sensitivity of 40.2%), with results providing no independent prognostic information at 30 and 90 days. Alternative studies have highlighted the high specificity of 94% for PCG detection of S3 for acute decompensated HF diagnosis in an emergency department [26]. Future studies should investigate whether an S3 is present before overt decompensation, and thus whether it could be used to trigger early intervention to prevent decompensation.

Similar to other modalities, PCG is vulnerable to motion, causing disturbances and noise in the microphone. One study also noted that the stethoscope bell became misplaced due to sweating whilst the patient was running [77], potentially limiting PCG for continuous measuring. Despite this, PCG has shown good accuracy in the ability to estimate BNP [78] and detect reduced ejection fraction [79]. In the context of decompensation, Susič et al. [27] applied a machine learning algorithm to heart sound recordings in patients with chronic HF and demonstrated that PCG could differentiate decompensated from compensated states with an accuracy of 72%, outperforming cardiologist interpretation (50%). These findings suggest that PCG may provide an accessible, non-invasive means of identifying early HF deterioration.

## 4. Wearable Sensors for Monitoring Fluid Status

Heart failure decompensation is primarily a fluid overload syndrome. Decreased cardiac output reduces renal perfusion and glomerular filtration rate, triggering sympathetic activation and renin–angiotensin–aldosterone system (RAAS) activation. This increases fluid and sodium retention, increasing intravascular volume and eventually expanding the interstitial volume [80]. Therefore, monitoring fluid status changes could provide early warning of HF decompensation, enabling timely intervention to improve patient outcomes.

Body weight monitoring has traditionally been the cornerstone of monitoring changes in fluid status in HF. However, weight monitoring has low sensitivity as gains are often gradual, with some patients admitted without weight gain, and others experiencing sudden ‘flash’ oedema [81]. Symptoms in the week before admission may have greater prognostic value, as intravascular fluid redistribution to the lungs causes pulmonary congestion and symptoms such as dyspnoea and exercise intolerance before weight gain occurs [82,83].

Monitoring lung fluid status could predict decompensation earlier than traditional methods. Pulmonary capillary wedge pressure (PCWP) assesses left ventricular filling through right heart catheterisation with balloon occlusion in a pulmonary artery branch to measure left atrial pressure [84]. However, this invasive procedure is inappropriate for widespread post-discharge monitoring, highlighting the need for non-invasive wearable-based DHTs to monitor lung fluid status.

### 4.1. Transthoracic Bioimpedance

Thoracic impedance estimates lung fluid volume by measuring electrical resistance across the chest, which decreases as pulmonary congestion develops. Implantable cardiac devices such as pacemakers, implantable cardioverter-defibrillators (ICDs), and cardiac resynchronisation therapy (CRT) systems can be equipped to measure thoracic bioimpedance by passing a subthreshold electrical current between device leads and the generator. Although reductions in impedance have been shown to precede HF hospitalisation by several days, randomised clinical trials have not demonstrated reductions in HF admissions or mortality when impedance-based alerts were used in isolation [85].

As implantable impedance monitoring requires device implantation, attention has shifted toward non-invasive wearable approaches for measuring thoracic bioimpedance and tracking fluid status in HF. Early pilot studies demonstrated the feasibility of vest-based thoracic bioimpedance monitoring in heart failure. For example, Cuba-Gyllensten et al. [86] tested a novel wearable vest on patients hospitalised with acute decompensated HF. The vest continuously measured transthoracic impedance as a surrogate for pulmonary fluid, successfully tracking changes in congestion during diuresis [87]. Building on this, Gastelurrutia et al. [28] showed that patients discharged with persistently low impedance values (<20 Ω) had significantly worse outcomes, with a higher rate of death from pump failure, whereas those achieving higher discharge impedance had substantially better survival [28]. The SENTINEL-HF study further tested this approach in 180 patients recently discharged after acute HF [29]. Daily 45 s vest measurements over 45 days were feasible and well adhered to, and declining or persistently low impedance values were associated with a higher risk of 30-day readmission. This supports the potential of wearable thoracic bioimpedance to identify patients at risk of early decompensation after discharge.

Researchers have also developed compact patch-based bioimpedance systems. One such study reported on a wearable multi-frequency impedance patch used in patients hospitalised with acute HF [88]. In this small prospective cohort study, a small wireless patch with chest electrodes continuously recorded thoracic bioimpedance throughout the hospital stay. The device successfully tracked fluid removal. As patients received diuretics, the patch showed increasing thoracic resistance (R at 80 kHz) corresponding to reduced lung fluid. Importantly, the study linked impedance trends to patient outcomes. Those whose impedance failed to rise (meaning persistent congestion) had markedly worse post-discharge prognosis. In fact, patients who did not show an increase in high-frequency impedance had significantly lower 30-day survival free from HF readmission compared to those with improved impedance. This demonstrated the feasibility and accuracy of a wearable patch for real-time fluid monitoring and showed that lack of impedance improvement can predict higher risk of adverse clinical events. The patch was well tolerated, supporting its clinical feasibility for inpatient monitoring of volume status.

### 4.2. Remote Dielectric Sensing (ReDS)

An alternate method of quantifying lung fluid volume is remote dielectric sensing (ReDS), which utilises low-power electromagnetic signals emitted and detected between two non-invasive electrodes attached to the chest [89]. This technology is similar to transthoracic bioimpedance but instead records the dielectric coefficient of the tissue, a measure of a tissue’s absorptance, reflection, and transmission of electromagnetic fields. Since water has a higher dielectric coefficient compared to air, fluid changes in the lungs will affect the calculated reading in patients with HF, providing an opportunity for this technology to monitor congestion. This technique has good accuracy with chest CT at quantifying lung fluid volume between patients with acute decompensated HF and non-acute decompensated HF [30] and high sensitivity and specificity with PCWP [31].

Several studies have utilised ReDS-derived fluid status information to predict HF decompensation and guide diuretic therapy. Bensimhon et al. [90] looked at whether ReDS-guided diuretic therapy could reduce rehospitalisation rates in comparison to standard therapy in patients initially admitted for decompensated HF. While ReDS was better at identifying patients at increased risk of rehospitalisation versus the control arm, ReDS-guided diuretic therapy did not affect the 30-day rehospitalisation rate. Conversely, Lala et al. [32] demonstrated that ReDS assessment and therapy in HF follow-up clinics could lower 30-day cardiovascular readmission rate. These studies suggest that ReDS is a useful tool for assessing lung fluid status, but its impact on reducing readmissions has been inconsistent. This may be because measurements are taken only at intermittent clinic visits, allowing changes in patient condition to go undetected between assessments.

While discrete or intermittent recordings have shown some benefit, continuous or daily recordings could detect fluid changes sooner. Using a continuous wearable ReDS vest, Amir et al. [33] observed that, in comparison to pre- and post-enrollment periods, ReDS-guided therapy could reduce the rate of hospitalisations by 87% and 79%, respectively, in patients initially admitted for acute decompensated HF. This suggests that the frequency of monitoring can greatly influence the success of the alert-guided therapies. Similarly, the ReDS-SAFE HF trial looked at using a ReDS-guided decongestion strategy, requiring a ReDS value ≤ 35% for discharge [91]. This study found that the treatment group had a lower rate of unplanned visits for HF, or HF-related hospitalisation, demonstrating the benefit of ReDS-guided systems for managing congestion post discharge. Sattar et al. [92], through a meta-analysis, noted that, irrespective of follow-up duration and whether inpatient or outpatient, ReDS could significantly reduce the rate of rehospitalisations. These results validate the use of wearable ReDS for alert-guided diuretic therapy, which is effective at reducing hospitalisations.

Similar to other technologies, ReDS has challenges which can limit its usability and accuracy. Despite taking around 3 min to record, it has high reliability even with single recordings [89]. However, ReDS is prone to changes with posture and time of day: it tends to be recorded sitting since legs raised or supine increases venous return, inflating ReDS recording [93], and tends to be greater in the morning, potentially due to increased sympathetic and RAAS activation [94]. Despite this, ReDS has strong potential in the world of wearable-based DHTs for remote monitoring of decompensation.

## 5. Multi-Sensor Systems and Algorithmic Prediction

Limitations of single-sensor systems have driven research toward multi-modal approaches (Table 3). Integrating multiple data streams provides a more comprehensive picture of physiological status, improving prediction of cardiopulmonary changes preceding HF decompensation. By combining sensor strengths and compensating for individual weaknesses, multi-modal devices may generate earlier, more accurate alerts and enable timely interventions that prevent hospitalisations. This synergy also broadens the range of physiological features available for machine learning algorithms, further enhancing detection accuracy.

This concept of combining multiple sensor inputs has been used with success in implantable cardiac devices. The MultiSENSE study, using data from CRT-D devices, incorporated heart sounds, respiration, thoracic impedance, heart rate, and activity to form a HeartLogic score, showing sensitive and timely prediction of HF events [99]. Numerous studies have examined ECG integration with mechano-sensing modalities, enabling complex parameter monitoring beyond heart rate. ECG combined with PPG and MCG waves provides cardiovascular electromechanical coupling information. Due to individual variation affecting PPG waves, ECG synergises with algorithms eliminating calibration requirements [100], particularly beneficial for remote monitoring where PPG calibration proves difficult. Similarly, MCG with ECG provides electrical baselines for cardiac time interval estimation [71]. Iqbal et al. [101] demonstrated practical remote monitoring feasibility using a wearable multi-modal device recording ECG, bioimpedance, and accelerometry data. The device operated continuously for 4.7 h with Bluetooth transmission to smartphones. Despite short battery life, dual-battery systems could enable continuous operation. This validates at-home HF decompensation prediction, though battery limitations require consideration.

The ZOLL LifeVest was able to combine ECG and accelerometry signals to derive ‘cardiac acoustic biomarkers’ for predicting HF decompensation in patients with HFrEF [97]. This multi-modal approach enabled detection of abnormal heart sounds (S3, S4), electromechanical activation time, and heart rate, which were incorporated into predictive algorithms. The algorithm achieved 69% sensitivity and 60% specificity for HF events, with a median prediction window of 32 days. Although the false positive rate was high, the negative predictive value of 94% demonstrates how multi-modal sensing can be useful for reliably ruling out impending HF events.

Other studies demonstrate benefits of combining cardiopulmonary data to directly monitor lung fluid states. Sanchez-Perez et al. [102] integrated transthoracic bioimpedance, computer-aided auscultation, multi-frequency impedance pneumography, and accelerometry in a pilot study, showing that the multi-modal system could estimate pulmonary fluid within physiological ranges and detect abnormal breathing patterns such as Cheyne–Stokes respiration. Extending this approach, the LINK-HF study [96] tested a chest-worn patch that continuously captured ECG, bioimpedance, accelerometry, posture, and temperature. Using machine learning analytics, the system predicted HF hospitalisation with 76–88% sensitivity and 85% specificity, generating alerts a median of 6.5 days before admission. Most recently, the BMAD trial [98] evaluated the ZOLL Heart Failure Monitoring System (HFMS), which integrates radiofrequency thoracic fluid monitoring with ECG and accelerometry. In patients discharged after a recent HF hospitalisation, HFMS-guided management achieved a 38% relative reduction in rehospitalisation at 90 days compared with usual care, alongside improvements in quality-of-life scores. Collectively, these studies highlight the promise of multi-modal wearables that combine electrical, mechanical, and fluid-status sensing to provide earlier and more accurate detection of impending decompensation.

Single-modality algorithms typically define thresholds for alert generation based on training data, indiscriminately alerting clinicians when thresholds are exceeded. However, machine learning algorithms have been developed that create predictive models assigning different weights to physiological data streams, enabling personalisation as algorithms learn individual baselines. Machine learning can detect complex non-linear patterns unnoticeable to clinicians, increasing predictive sensitivity. The MUSIC study designed and tested a monitoring algorithm for early acute decompensation detection [95], integrating bioimpedance, ECG, and accelerometry. This study compared single- and multi-parameter algorithms, finding that, whilst single-parameter algorithms achieved sufficient sensitivity (>60%), high false-positive rates limited usability. Multi-parameter algorithm research examined three permutations, demonstrating acceptable sensitivity and specificity when combining a fluid index and breath index with personalised patient characteristics (age, weight, sex, height). The validation cohort achieved 63% sensitivity, 92% specificity, and 0.9 false positives per patient-year, meeting performance endpoints.

## 6. Challenges, Limitations, and Future Directions

Before wearable-based DHTs predicting HF decompensation achieve widespread outpatient use, several challenges must be overcome (Table 4). Two main objectives are essential: firstly, accurate detection with sufficient pre-symptomatic warning to clinicians; secondly, adequate intervention time to improve patient outcomes compared to those without early-warning systems. Limiting unnecessary alerts and false positives is crucial to prevent increased clinician workload and excessive patient stress. For improved accuracy, devices should provide personalised alerts using appropriate algorithms for all demographics, including those with complex comorbidities and varied body profiles. High patient adherence is essential in settings with minimal clinical oversight, often dependent on device size, complexity, and connectivity amongst potentially digitally inexperienced populations. Devices should therefore be user-friendly, requiring minimal patient or caregiver training to improve compliance. Beyond patient factors, significant considerations include data management, security, and integration into existing systems. Regulatory approval requirements further complicate routine clinical implementation.

### 6.1. Accuracy and Standardisation

Several studies mentioned above have demonstrated good accuracy for predicting impending decompensation: the LINK-HF study achieved a sensitivity between 76.0% and 87.5% with an 85% specificity [96], and the MUSIC trial obtained up to a sensitivity of 63% and a specificity of 92% [95]. The LINK-HF trial was also capable of generating alerts before hospitalisation with a median time of 6.5 to 8.5 days. However, many studies lack sufficient sensitivity for decompensation episodes, with high rates of false positives, such as the Erath et al. [97] study using the ZOLL Lifevest. From a practical perspective, reporting false-positive and false-negative rates per patient-year provides the most meaningful measure of clinical reliability, directly influencing both patient trust and clinician adoption of multi-sensor monitoring systems.

There is also a lack of standardisation between these studies, each using unique predictive algorithms, establishing different thresholds and weights of physiological factors in their models. Larger-scale RCT studies may improve the current models and provide sufficient patient data to train models representative of the HF population. For example, Fokkema et al. [46] demonstrated a large variability in the estimated step counts between different devices, suggesting the need for standardisation to validate model predictions.

### 6.2. Data Interpretation Methods

Sufficient evidence supports multi-modal over single-modal devices, improving predictive model strength by considering various physiological parameters altered preceding decompensation. However, the true benefit lies in using algorithms to manipulate and identify complex patterns, extracting additional features from otherwise simple data metrics, such as Figo et al. [103], who looked at extracting movement type and quality from basic accelerometer data.

The evolution from TIM-HF to TIM-HF2 demonstrates algorithm-guided interpretation potential versus clinician-only data analysis. The Telemedical Interventional Monitoring in Heart Failure study (TIM-HF, [104]) transmitted daily physiological data to 24/7 telemedical centre clinicians. Compared to usual care, remote monitoring showed no significant benefit in reducing all-cause mortality or cardiovascular death/HF hospitalisation. The subsequent TIM-HF2 study [105] transmitted similar parameters but utilised rule-based alert systems flagging higher-risk patients. The algorithm combined patient-transmitted data with clinic-obtained blood biomarkers to characterise risk, enabling patient prioritisation and prompt medication uptitration. Remote monitoring reduced days lost to unplanned cardiovascular hospitalisations and all-cause mortality versus usual care, although no effect was seen on cardiovascular mortality.

This suggests that, despite rule-based risk-stratifying algorithms, true benefit lies in ‘intelligent’ algorithms identifying decompensation patterns for earlier alerts. Regardless, these results suggest remote monitoring improves patient outcomes and validates future research utilising machine learning and continuous multi-modal devices, potentially improving predictive capabilities and further reducing hospitalisations and cardiovascular mortality.

### 6.3. Patient Usability and Adherence

From traditional Holter monitors, focus has shifted to simplifying devices, reducing size, and improving comfort to enhance adherence. Major usability limitations include device size, conductive gel requirements/adhesive reactions, comfort (related to attachment site), user interface complexity, and data uploading challenges.

One validation study examined Fitbit device usability and adherence for HF self-care support [106], noting strong motivation to use the system (Fitbit plus digital health tool), supported by device comfort. However, other devices show poorer adherence, particularly waist-worn devices (including vests) removed during sleeping/showering and forgotten afterwards. ECG technology advances enable dry electrodes, eliminating inconvenient and potentially irritating gel requirements [68]. Battery life limitations can cause extended recording gaps if patients forget charging/replacement, limiting clinician data transmission. Focus should therefore be on extended battery life with user-friendly charging solutions to maximise usage. For devices requiring Bluetooth connections or periodic data uploading, processes should be streamlined to minimise patient involvement. For older patients with HF potentially lacking digital awareness, simplified user interfaces and reduced system interaction requirements can maximise data transmission. This supports exploring existing products like wrist-worn smartwatches with simple interfaces and appropriate battery life for HF decompensation prediction.

### 6.4. Data Management and Security/Integration

Another important consideration is patient data management and integration into existing clinical systems, emphasising data confidentiality and security. Platforms should consider secure portal uploads with appropriate access privileges preventing unnecessary and inappropriate patient data access. Given massive daily data volumes recorded and transmitted, questions arise regarding data manipulation and determining valuable versus extraneous information. Serrano et al. [107] reviewed healthcare professional challenges, citing concerns that remote monitoring increases workload with integration difficulties into existing medical records. Legal and security constraints on patient data sharing further complicate platform integration [108]. Additional concerns include remote monitoring inaccessibility for lower-income populations, potentially widening healthcare disparities.

Effective integration of alert management into existing heart failure care pathways is essential for clinical impact. In practice, structured escalation involving heart failure specialist nurses or telemonitoring teams must enable timely response to alerts, facilitating early therapy adjustment and patient support. Such coordinated models are important to translate predictive monitoring into improved outcomes and sustained clinician and patient confidence.

Whilst remote monitoring may reduce economic healthcare burdens from HF hospitalisation costs and increased clinic visits during exacerbations, devices remain expensive for patients with unclear financing or reimbursement processes. These attitudes are supported by studies examining economic burdens of other remote monitoring strategies, including continuous glucose monitors [109] and cardiovascular disease management [110]. Targeted studies into remote monitoring economics for HF decompensation could establish significant economic benefits for reducing hospitalisations and whether reimbursement or subsidisation schemes could improve access.

### 6.5. Device Readiness and Regulatory Considerations

Fundamental to establishing safety and ensuring effectiveness, devices for clinical use outside experimental studies must achieve appropriate regulatory acceptance. Devices can be assessed against Medical Device Readiness Level (MDRL) criteria, establishing development stages, with final approval from the European Union’s Medical Device Regulation (MDR) and U.S. Food and Drug Administration (FDA). The MDR and FDA use guidelines to classify and regulate potential medical devices. Scholte et al. [111] reviewed studies using accelerometers, transthoracic impedance monitors, PPG, and ECG, assessing device readiness and study outcomes. Only eight identified studies were RCTs, with none testing device effectiveness in large randomised trials. The authors noted this lack of robust evidence, contributing to barriers integrating these devices into routine HF management.

Interestingly, Scholte et al. [111] differentiated between single-measurement techniques and multi-modal devices. Most single-modal devices were in feasibility testing stages (MDRL 6), whilst multi-modal devices achieved higher MDLR ratings for certain metrics. These include the ZOLL Lifevest achieving MDLR 9 for heart rate function and MDLR 7 for the Fitbit Charge HR. Despite some developmental advances, others remain in prototyping stages (MDRL 3). Scholte et al. [111] highlight that, whilst some devices are more developed, they are often designed for other uses and populations (only two FDA-approved wearables were designed specifically for HF remote monitoring). Despite being consumer-grade devices, they lack FDA or MDR approval for HF patient monitoring. These devices’ true potential remains unclear; however, previous research warrants further large-scale RCTs to verify non-invasive wearables’ value in predicting HF decompensation.

## 7. Conclusions

Many studies have shown the potential of non-invasive devices to monitor physiological features, with the capability of predicting decompensation. Some trials have also demonstrated the utility of wearable-based DHTs in HF remote monitoring and therapy changes, in turn reducing hospitalisations and improving patient outcomes. Research demonstrates the improved accuracy and capability of a multi-modal approach, however, issues regarding standardisation and the use of different algorithms trained on different datasets have led to a large variation in the predictive ability of these models. Beyond issues of accuracy, there remain substantial challenges in usability, patient adherence, integration with existing healthcare infrastructure, and regulatory oversight, all of which hinder the routine implementation of wearable-based DHTs. Despite this, current evidence demonstrates multiple physiological parameters with which monitoring with non-invasive wearables may enable remote monitoring and hence HF decompensation prediction. Ultimately, large-scale randomised controlled trials are needed to determine the true benefits and considerations of using non-invasive remote monitoring in a targeted HF population.

## Figures and Tables

**Table 1:** Physiological parameters targeted by wearable modalities.

Physiological Parameter	Wearable Modality	Physiological Rationale
Physical activity and exercise tolerance	Accelerometer, actigraphy, IMU	Reduced activity, increased sedentary time, disrupted circadian rhythm often precede HF decompensation
Heart rate/HRV	PPG, ECG	Circadian rhythm disruption, reduced HRV and rising resting HR indicate autonomic imbalance and impending deterioration
Cardiac electrical activity	ECG (patches, watches, vests)	Abnormal QRS, PR intervals, arrhythmia burden, and reduced QRS amplitude correlate with worsening HF
Cardiac mechanics	Mechanocardiography (MCG), phonocardiography (PCG)	Altered vibrations (S3, reduced stability of S1), reduced reserve detectable via seismocardiography
Pulmonary congestion	Thoracic bioimpedance, remote dielectric sensing	Falling impedance or rising dielectric coefficient reflects lung fluid accumulation

**Table 2:** Key clinical evidence for single-modality wearables.

Modality	Patient Cohort	Key Findings	Limitations
Accelerometry (step count)	Patients with HF in Japan [18]; rural US cohort [19]	Decreased step counts can predict HF diagnosis and cardiac mortality; feasibility established but adherence variable	Adherence issues, device variability
Accelerometry (sedentary time, activity intensity)	UK Biobank (89,000) [20]; post-discharge HF [21]	>10.6 h sedentary time ↑ HF risk; decompensated HF patients spent only 9% awake time non-sedentary	Non-specific (affected by comorbidities)
Photoplethysmography (PPG)	Patients with HF vs. non-HF [22]	HRV and pulse interval analysis feasible; pilot AUC = 0.85–0.92 for distinguishing HF vs. controls	Motion artefact, daytime data gaps
ECG (single lead, mobile)	Acute HF [23]; wrist-worn and vest wearable studies [24]	ECG features predicted NT-proBNP and 30-day mortality; CNN model 91.6% accurate for NYHA classification	Limited validation; device variability
Mechanocardiography (MCG)	Acute HF (admission vs. discharge) [25]	Root-mean-square SCG/GCG signal strength tracked congestion and haemodynamic	Limited to research; not continuous yet
Phonocardiogram (PCG)	ED patients with dyspnoea [26]; chronic HF [27]	S3 detection specificity 94% for acute HF; ML models have 72% accuracy for decompensated vs. stable HF	Motion artefacts, low sensitivity of S3
Thoracic bioimpedance	Patients recently discharged with HF [28,29]	Persistently low impedance predicted readmission	High false positives when used alone
Remote dielectric sensing (ReDS)	Acute HF and follow-up cohorts [30,31,32,33]	ReDS-guided therapy reduced readmissions in some trials; strong correlation with PCWP and CT	Posture-dependent, cost, clinic-based in most studies

**Table 3:** Multi-sensor systems and algorithmic approaches.

Trial	Sensors Integrated	Predictive Performance	Median Lead Time	Strengths	Weaknesses
MUSIC study [95]	Bioimpedance + ECG + accelerometry	Sensitivity 63%, specificity 92%	Not specified	Reduced false positives vs. single sensor	Limited by dataset size
LINK-HF vital patch [96]	ECG + bioimpedance + accelerometry + posture + temperature	Sensitivity 76–88%, specificity 85%	6.5 days before admission	Continuous monitoring; personalised ML	Battery life
ZOLL LifeVest [97]	ECG + accelerometry + acoustic biomarkers (S3, S4, EMAT)	Sensitivity 69%, specificity 60%, NPV 94%	32 days	Comfortable, commercial device	High false positives
BMAD trial—HFMS [98]	Radiofrequency thoracic fluid + ECG + accelerometry	38% reduction in 90-day readmission	Days–weeks	RCT evidence, improved QoL	Expensive, new tech

**Table 4:** Challenges and barriers to implementation.

Domain	Key Challenges	Future Directions
Accuracy and standardisation	Variability between devices, lack of consensus thresholds, motion artefacts (e.g., PPG)	Standardisation protocols, larger RCT validation, AI-driven calibration
Data interpretation methods	High false positives, complex raw data, clinician burden	Multi-parameter ML, personalised baselines, adaptive alerts
Patient usability and adherence	Poor compliance with vests/waist-worn devices, skin irritation, charging requirements	Smaller devices, wrist-based systems, improved battery life, dry electrodes
Data management and security/integration	High data volume, clinician workload, EHR interoperability issues	Streamlined dashboards, automated triage, secure cloud storage
Equity and access	High cost, limited reimbursement, digital literacy barriers	Subsidies, payer engagement, simplified interfaces
Device readiness and regulatory considerations	Few FDA/MDR-approved devices specifically for HF monitoring	Larger RCTs, MDRL/FDA pathways tailored to wearables

## Data Availability

No new data was generated in this work.
